# The Hospital. Nursing Section

**Published:** 1905-11-04

**Authors:** 


### The Hospital.

### Nursing Section.

Contributions for this Section of "THE HOSPITAL" should be addressed to the EDITOR, "THE HOSPITAL" NURSING SECTION, 28 & 29 Southampton Street, Strand, London, W.C.

No. 997.—VOL. XXXIX. SATURDAY, NOVEMBER 4, 1905.

### Notes on News from the Nursing World.

### THE QUEEN AND THE ARMY NURSING BOARD.

In his address at Brighton last Friday on the Army Medical Service, Sir Frederick Treves referred to the profound interest which the Queen, as President of the Army Nursing Board, takes in the questions that come before the Board. Sir Frederick states that not only does her Majesty attend the meetings, but the minutes of all the meetings are forwarded to her, and she generally sends for someone to discuss the points. There was not, he said, a single movement in the development of the nursing service that the Queen had not been familiar with, and in the organisation of which she had not taken an active part. We are glad to have the authority of Sir Frederick Treves for the intimation that the nursing service is very well equipped, and "that no fewer than 260 nursing sisters have been added to it since the Queen insisted upon its being put in order."

### THE SITUATION AT MILLBANK HOSPITAL.

So far, the situation with regard to the Nurses' Home at Millbank Military Hospital is apparently unchanged. The building operations are proceeding in the same way as before, and no intimation that fresh plans are being prepared has been received. There seems to be a serious probability that, at the best, the story of the hospital itself will be repeated in the case of the Nurses' Home. When the members of the nursing staff were posted to Millbank it was pointed out that several important alterations would have to be effected before the work could be carried on in a satisfactory manner. Notwithstanding this, patients were sent to the building, and now that most of the wards are full, these alterations have been begun. Not only is this mode of procedure, which affects the building from roof to basement, causing the greatest inconvenience to everyone, but it will involve an outlay of several thousand pounds. The repetition of the same policy in regard to the Nurses' Home will be far more expensive than enlarging the bedrooms, or increasing the accommodation, whilst the Home is still in an unfinished condition.

### TO PENSION FUND NURSES.

We are asked by the Hon. Secretary of the Junius S. Morgan Benevolent Fund to remind those policy-holders who have forgotten, that their contribution of 1s. to the fund is due, and is very much needed. This yearly contribution to the Benevolent Fund was suggested by a policy-holder about nine years ago. But, unfortunately, the amount subscribed shows that not nearly all those who belong to the Pension Fund help to provide, by this small donation, for their fellow-nurses who are in distress. The Committee of the Benevolent Fund wisely give assistance according to the funds at their disposal. They do not believe in the principle of going into debt in order to make an effective appeal. They hope, therefore, that all the policy-holders will support them in their work, and enable them to give to every nurse the amount of assistance necessary, without fear that applicants late in the financial year will find the coffers empty.

### A CHRISTMAS TAX ON NURSES.

Two correspondents write to us from different parts of the country protesting, at an opportune moment, against the continuance of the custom of giving presents at Christmas to members of the hospital staff, from the matron downwards. We say without hesitation that the custom is more honoured in the breach than in the observance. It is due, no doubt, to the best of motives, and we should not like to discourage manifestations of the desire to promote good fellowship. But this, we are quite satisfied, can be fostered without maintaining what one of our correspondents describes as wholesale taxation. We therefore earnestly hope that matrons, who are not by any means the least sufferers from the system, though they may not feel the pinch so severely as the poor nurse, who has relatives even poorer needing her help, will seriously consider the matter, and decide to exercise their authority. We agree with the sister who affirms that the matter is, practically, at any rate, in the hands of the matrons, and we think that if they incur a little passing unpopularity by putting a stop to the tax, they will earn a great deal of gratitude.

### THE ADA LEWIS INSTITUTE.

We regret that the indisposition of Mrs. Lewis-Hill prevented the opening of the Ada Lewis Nurses' Institute at 62 Oxford Terrace on Thursday last. But we understand that the work of the Institute was commenced as arranged, and we hope that the formal ceremony is only delayed for a little while. Meanwhile, we notice that the movement has attracted attention in Scotland, and is provoking criticism. While the value of Mrs. Lewis-Hill's experiment is fully recognised, it is maintained that if provision of skilled nursing at rates attainable by persons of moderate means is to become general throughout the kingdom, it must be based on other foundations than charity or philanthropy. There is a good deal of force in this argument, but before attempting to form co-operative societies to employ a certain number of trained nurses, which is suggested as a means of supplying nurses for persons of limited means, it might be well to await the result of the experiment for which the community is indebted to Mrs. Lewis-Hill. It should prove an admirable object-lesson.

### THE POWER OF THE NURSE

One of the best known of the London clergy, who has been interviewed on "The Church and Christian Science," being asked whether it was his impression that the tendency to encourage the visitation by the clergy of the sick is less than it was, replied in the affirmative, so far as cases of acute sickness are concerned. "This is probably," he continued, "one of the results of the more careful system of modern nursing, which lays stress on the importance of keeping the sick-room quiet. The old idea was that the friends of the sick person should be allowed to crowd into the room. Now the nurse shuts them out, with good results in the main. The nurse has, therefore, become an exceedingly powerful force in regulating the arrangements of the modern household when there is illness in the home; and it often practically rests with her, more than with the doctor, to determine whether the patient shall be visited by the parish priest." This is a remarkable recognition of the power of the nurse, but it fastens upon her a great obligation, which requires to be discharged with a keen sense of responsibility. In the same interview it is affirmed that Christian Science shuts the door in the face of the doctor and the nurse, and that it "occasionally happens that scientific nursing shuts the door in the face of the priest," the attitude of one being described as representing a revulsion from that of the other. However that may be, the nurse who can send for or prevent the attendance of the parish priest should possess some of the highest qualities which can belong to a woman.

### THE POOR-LAW SUPERINTENDENT NURSE.

At the last meeting of the Barnsley Guardians Mr. P. H. Bagenal, Local Government Board Inspector, alluding to the resignation of the superintendent nurse at the workhouse after a stay of three months, recognised the need of defining the relative position of the master and matron in regard to the superintendent nurses. Mr. Bagenal says that the Local Government Board are inquiring into the matter. This is satisfactory as far as it goes, and we conclude that the investigation, which seems to have been proceeding ever since the issue of the report of the Departmental Committee, is at length about to be followed by action.

### CONFERENCE OF QUEEN'S SUPERINTENDENTS.

The subjects to be discussed at the Conference of Queen's Superintendents in the Northern and Southern Counties to be held in London on November 29 are, "How Inspection may be Made of the Most Use," "How Best to Utilise Outside Help," and "The Relation of Queen's Nurses to the Local Supervising Authorities under the Midwives Act." The chair, as on past occasions, will be taken by Miss Rosalind Paget, and, as intimated in our issue of September 30, the presentation of a cheque to Miss Peter will take place on the occasion. An address will also at the same time be handed to Miss Peter with the names of the subscribing nurses, not far short of a thousand. The superintendent present who has been longest in the service of the Jubilee Institute will make the presentation.

### TYPHOID FEVER AT PORTSMOUTH PARISH INFIRMARY.

Last week we announced the death of a probationer at Portsmouth Parish Infirmary from typhoid fever. We regret to learn that another nurse attached to the Infirmary, Miss Amelia Hill, has succumbed to the same disease. She had been nursing several cases of typhoid. This second death suggests that proper precautions were not taken. There is but little more danger in nursing typhoid, with the observance of well-recognised conditions, than in the nursing of small-pox by persons who have been vaccinated.

### NUNS AND TRAINING.

It is stated that "nine of the nursing sisters of St. John of God, at Wexford, have successfully passed their examination in elementary anatomy and physiology, and in medical, surgical, and fever nursing, having completed a course of instruction given by an experienced nurse from London, who has had many years' experience in training probationers and nurses in public hospitals." The training given by a nurse from London, however experienced, and an examination by two medical men, and the instructors of the matrons at the Waterford District Hospital, cannot be regarded in the same light as a course of three years' training in a recognised school; but this is a step forward in the right direction, at least so far as nuns in Ireland are concerned.

### THE INSTRUCTION OF IGNORANT MOTHERS.

At the Conference of Women Workers, held in Birmingham last week, it was admitted that ignorance on the part of the mothers was largely responsible for the heavy infant mortality. Several speakers strongly condemned the practice of turning young mothers out of the workhouse with no knowledge whatever of how to treat their babies, because from the time the child is born till it leaves the institution it is fed, washed, and cared for by the attendants. The result is that as the mother does nothing she leaves knowing nothing, and when the same woman later enters the infirmary again and is asked about her first child, the inquirer is usually told that the baby is dead. It is a matter for regret that the mothers, upon their return to their own homes, should have learnt so little about the treatment of their infants; but it is impossible, as matters now stand, for an alteration to be effected. The lying-in wards of our hospitals and infirmaries are, as a rule, not sufficiently staffed to allow of any of the nurses being told off to daily instruct mothers who will soon be leaving the wards, which would be the only way of meeting the case.

### THE PRIVATE NURSING QUESTION AT SWANSEA

Another attempt has been made to continue the private nursing at Swansea Hospital, but after an animated discussion, in which several members of the Board took part, the Chairman gave the casting vote against a resolution in favour of the rescission of the motion agreeing to the suspension of the department. We regret persistence in a policy of negation, and we advise the advocates of the continuance of the private staff to persist in agitating the question until it is disposed of one way or the other.

### MARYLEBONE NURSES AND THE PENSION FUND

On Tuesday, November 14, Mr. Louis H. M. Dick, Secretary of the Royal National Pension Fund for Nurses, will give a short address to the nurses of St. Marylebone Infirmary at 5 p.m. Any member of the nursing profession will be cordially welcomed.

### NURSING ORDERLIES AND MASSAGE.

We understand that the Incorporated Society of Trained Masseuses, having been applied to by the War Office to organise an examination in massage of nursing orderlies of the Royal Army Medical Corps, an examination was recently held at Netley Hospital, when 17 candidates satisfied the examiners. Now that the powers of the Society have been extended by the Board of Trade to meet this application, it has been arranged that in future the examinations will be held twice yearly at Millbank.

### THE ROYAL BRITISH NURSES' ASSOCIATION IN THE TRANSVAAL.

A protest is made in a Johannesburg paper against the proposed acceptance for registration by the Medical Council of the Transvaal of the certificate granted by the Royal British Nurses' Association, of which a branch has just been formed in South Africa. The protest is based on the ground that the certificate in question is granted to women who have worked in hospitals for one or two years, and to some with fever training only. Meanwhile, it is stated that Mrs. Temple Mursell, Lady Consul for the Transvaal, has practically succeeded in forming a local committee of medical men and heads of nursing institutions.

### NURSES' HOSTEL COMPANY.

The eighth annual general meeting of the Nurses' Hostel Company was held on Tuesday, when the report of the directors was adopted. Regret was expressed at the death of Miss Mary Hope, who had been a director from the formation of the company. Miss F. G. Cooper, formerly Matron of the Victoria Hospital for Children, has been selected to fill the vacancy. The continued appreciation of the Hostel by the nurses is shown by the fact that the number of visits to the two blocks during the year was 208 in excess of the previous twelve months; and as to its financial position, a dividend of 4 per cent. was declared, £80 was added to the sinking fund, £50 placed to the credit of reserve, with a small balance carried over.

### EXETER NURSES AND DIRTY PATIENTS.

In reporting on the examination of several of the probationers of the Royal Devon and Exeter Hospital, Dr. W. Hale White, of London, says that they did their work extremely well, and that the only weak point he detected in their knowledge reflects a little to the credit of Exeter, "for they were not quite as familiar as they should be with the best way of cleansing a thoroughly dirty patient, the person usually styled a verminous person." All the nurses, he states, had evidently been most carefully trained in practical details. The highest marks were obtained by Nurse Risk, who got 1,040, and Nurse Ballantyne, who got 1,032. As the result of this report Nurse Risk will receive a gold medal and Nurse Ballantyne a silver one.

### PECKHAM NURSING ASSOCIATION.

The fifth annual meeting of Peckham Nursing Association was held last week. Mr. Alderman Goddard Clarke, the Chairman, reported that during the past year 2,550 visits were paid to 133 poor patients in their own homes. Since the Association was inaugurated nearly 10,000 visits have been paid to 520 patients. Early this year a permanent Nurses' Home was started at 91 The Rye, as a centre to which, at any time, the local medical men and clergy can send for nursing assistance. The Association has been compelled to extend its field of operations, and is in sore need of funds, for which an urgent appeal is being made to the residents of the locality.

### THE VALUE OF A THIRD NURSE.

The Hanley Nursing Society have an annual sale of work, which produces on an average about £130 towards the £450 required for the support of the institution. At the opening ceremony this year the President stated that in spite of the decision meeting with some criticism, they determined in 1903 to retain the services of a third nurse. He thought that the greatness of the need had been demonstrated by the fact that with a third nurse 2,000 more visits had been paid to the sick poor than had been possible when the Society had only two nurses.

### OUR CHRISTMAS DISTRIBUTION.

We have received contributions to our Christmas distribution from Miss Sharwood, Hove; and two parcels from Miss Taylor, Dulwich. All parcels should be addressed to the Editor, 28 and 29 Southampton Street, Strand, London, W.C., with "Clothing Distribution" written on the outside.

### SHORT ITEMS.

There were no fewer than 50 candidates for the post of Lady Superintendent of the York Home for Nurses, the choice of the Council falling upon Miss Margaret Morgan, who, from September 1901 to March, 1905, was Matron of Addenbrooke's Hospital, Cambridge.—A correspondent sends us an effective postcard portraying the matron and her three nurses grouped together under the trees in the garden, which she concisely describes as "the *entente cordiale* between the Matron and her nurses at Atcham Workhouse."—Last week Dr. Jellett, Gynæcologist and Obstetrician, Dr. Steevens' Hospital, gave an interesting address on "Emergencies" to the members of the Irish Nurses' Association. There was a large attendance of the members, and at the close of the lecture a hearty vote of thanks was accorded to him.

### The Nursing Outlook.

**"From magnanimity, all fear above;
From nobler recompense, above applause,
Which owes to man's short outlook all its charm."**

### EXISTING REGISTERS OF TRAINED NURSES.

For many years the nurse-training schools which have any claim to such a title have kept a very complete and accurate register of the training and conduct of the probationers, sisters and nurses during their residence at the hospitals in question. As a matter of business such a register is essential to the proper conduct and administration of every nursing department in a hospital, and the same remark applies to nursing homes working under a committee which supply nurses to the public. We take it that in fact, where such registers have not been kept, it is largely due to the circumstance, that few women have yet had sufficient business training, and that no adequate methods at present exist for the complete instruction of those who aspire to become the matron or lady superintendent of a great institution. The issue of the complete report and evidence of the Select Committee on the Registration of Nurses provides every one interested with a form of register of the best kind. Those who will refer to Appendix 11, page 193, will find a specimen of the register of nurses kept by King's College Hospital. That register sets forth fully particulars of the circumstances, previous history, and experience of each probationer. It records the work she has actually done during the three years' period of training, together with a record of all her subsequent work should the nurse remain in the service of the hospital. It embraces a copy of the certificate of training, and the degree of efficiency to which each certificated nurse has attained, the results of each examination, and the home sister's report. It further provides space for a record of the subsequent work and appointments of each certificated nurse who may leave the service of the hospital.

Here then is a complete and excellent form of register which any institution not at present keeping such a book may readily adopt without difficulty or delay. As we recently said, it is of the first importance, now that every nurse-training school has fallen into line with the three years course, that they should lay themselves out to facilitate, to the utmost, the co-operation of all nurse-training schools. With this object the existing registers will no doubt be closely overhauled to see that they are made as perfect and complete as possible. It is no use preaching to the converted, but it is still true that, although the nurse-training schools do in fact keep a very complete and accurate register, there are still some hospitals, and amongst them a few large provincial hospitals, which do not possess accurate records of the nurses who have been in their employ, who may have had the three years' certificate. In a few cases of general and county hospitals and infirmaries no adequate records have been kept, and it may be possible that in one or two cases no such records exist. In these circumstances we would make an urgent representation to the committee and officials of all the hospitals which employ probationers, or are dependent upon the training of other institutions for the supply of nurses which they need, to look into this question of a register, and to see that steps are taken to at once set up a book, which contains an accurate record of all the nurses at present engaged at the institution.

It is desirable that something more than this should be done in the direction of providing, as in the case of King's College Register, that the after service of every nurse who may at any time enter the service of a large hospital, shall be recorded with as much accuracy as possible in the hospital books. Such a step is desirable in the interests of the institution and of the nurse herself. We have always advocated the cultivation of the best relations possible between employers and employed. Their interests should be and are largely identical, and any one who makes it their business to set the nurses against the authorities for whom they work renders an ill service to both, and is not entitled to be regarded as the friend of either party. It is to the interests of both, and necessary for the protection of the public, that every matron should keep in touch, as far as possible, with all nurses who may pass through her hands in the course of her working life. When the matron is promoted from one hospital to another, her successor, by keeping up the records, as the matron herself will keep the records in the new institution to which she has been appointed, co-operates by keeping touch with all the nurses on the register, whether then in the service of the hospital or not, and so renders substantial service to the public which cannot fail to be appreciated—a course which is certainly necessary and justifiable in the circumstances.

Committees who aspire to administer the affairs of the hospital under their control in the best possible way, must necessarily keep a sharp eye on the books and records of their institution. It is a part of their duty to provide that the register of nurses shall be in the proper form, and that it is kept up to date by the entry of all material particulars, especially in those institutions where probationer nurses are received for training. We have often heard committee-men say, that the work would be much more interesting, if they could devote themselves to one special department or branch. May we suggest, that no better service could be rendered by one member of each committee, than to devote his or her time to the supervision and accurate maintenance of the records and registers, which should be kept in connection with the nursing department of every well administered institution? We have often been attacked for upholding the general efficiency of the nurse-training schools and their registers, and we hope that committees and officials will now cordially co-operate in securing that each institution shall possess adequate registers, and that all records and registers shall henceforth be properly and accurately maintained.

### Laryngeal Diphtheria.

By Mrs. HICKS, Formerly Matron of the Evelina Hospital for Sick Children.

### II. INTUBATION AND TRACHEOTOMY.

(*Continued from page* 43.)

Intubation of the larynx has been practised for many years, but it is only since the introduction of antitoxin in 1894 that it has come into vogue so fully for diphtheria. O'Dwyer, an American surgeon, has invented a set of intubation instruments which are much used, and the only things required in addition to the box of these instruments is some silk thread, a tongue depressor, linen rags, sponge holders and swabs, a bowl to receive the latter, a small piece of strapping, and a Belocq's sound. The intubation set consists of a gag, an introducer, to which can be screwed any of the pilots or obturaters of the different tubes—the tubes vary in size, and are graduated according to the age of the patient—an extubator, and a gauge for measuring the tubes. When the child is to be intubated, the nurse asks the doctor which tube he will use, and accordingly gets ready the tube required, threading it evenly through the eye at the broad part with a double piece of silk, hanging loose at the far end with the pilot inside screwed perfectly straight to the introducer.

The next thing is to find out whether the child is to be intubated in its cot. In this case the hands are tied to the sides of the bed, the nurse standing on the left side of the patient, and with the right hand steadying his head in an absolutely straight position by pressure on the forehead, while the other hand is ready to hold the gag which the doctor will fix between the left jaws of the child, so as to have the right side free for his manipulations. It is useful to have a second assistant who fixes the child's legs by holding them down above the knees; if, however, this help is not available the legs should be tied, fully-extended, to the foot of the bed, passing the bandages above the ankle. Most surgeons prefer this position to placing the patient on the nurse's lap, because, although the child can be immobilised to a certain extent by rolling it up firmly in a blanket, the operator has not the same advantage of looking down and keeping in the middle line. The nurse holds the child's head pressed back against her shoulder, and with her other hand steadies the legs. A second assistant is generally required for the gag.

The great point which the nurse must remember is to have the child, lying rigidly straight, the mouth well open and the gag properly secured. The doctor passes his left index finger into the mouth, feels for the top of the larynx, he then runs the tube on the introducer along the index finger, keeping the handle depressed and absolutely in the middle line—this can only be done if you hold the head straight—until the end of the tube lies in front of the left index. He then elevates the handle of the introducer to bring the tube into the long axis of the trachea, and the tube slips into the larynx with its beak between the arytænoid cartilages. The introducer with the pilot are quickly withdrawn while the tube is being steadied with the left index. With the gag still in the mouth the double thread is tied behind the ear and secured in front of the ear by a piece of strapping. If there is any danger of the string being bitten through some doctors prefer to pass the string through the nose with a Belocq's sound, then securing it behind the ear.

To the question of the tube being in the right place, the patient will respond clearly by the very characteristic rattling intubation cough, immediate relief of the distress, and improvement of colour. The doctor will, as a rule, swab out the throat thoroughly before removing the gag, and it is useful for the nurse to watch him do it, and observe how carefully he avoids catching in the string. The nurse may have to repeat this swabbing over and over again, and she does it by inserting the tongue depressor well back until it touches the posterior part of the tongue, and the child is obliged to open the mouth, and then swabs out carefully until the part is free of discharge. This is almost the only local nursing to be done, but may have to be repeated every few minutes for hours, or even days, until the rattling ceases.

As soon as the doctor has finished the nurse should get her patient comfortable, release the legs, and as tying the hands is a very harsh proceeding, put on a pair of arm-stays which will immobilise the elbows, and while thus preventing the patient from touching the tube, will allow him to play with toys, and thus be much happier. Be most careful to fix these straps so that they reach midway between elbow and armpit, so that the child is unable to bend the arm. The irritation caused by the string in the mouth is very great, and the patient naturally tries to relieve this if you give it the slightest chance, and the results may be disastrous. Now clean your introducer, and pilot and lay them in a dish on the bedside locker with the gag, a reserve piece of silk, some sponge-holders with swabs, linen rags, a probe to clean the tubes with, and some warm saturated solution of bicarbonate of soda for the same purpose. If a duplicate tube exists have it ready on the introducer for an emergency.

The child may now either go on well, only requiring occasional swabbing, or he may cough out the tube. Then ring your bell loudly, quickly clean the expectorated tube by passing rags soaked in the lot. sod. bicarb. through it on the end of the probe, thread the tube with the fresh piece of silk, insert the pilot on the introducer, tie patient's feet and hands, and by the time the doctor comes up be ready to hold the child for re-intubation. A third thing may happen. The child, though apparently at first relieved, may gradually become cyanosed and dyspnœic again. In this case ring your bell. But if the dyspnœa should become suddenly extremely urgent, so that you expect the tube to be entirely blocked, you must, after ringing the bell, remove the tube yourself. This is quite easy to do, if the string has been passed through the mouth. Simply introduce the tongue depressor well back with the left hand, and with the right pull gently on the string until the tube glides out of the larynx. Do not, in your excitement, jerk it out quickly, for you may, by using force, scrape and injure permanently the delicate structures of the larynx. If the string has been passed through the nose, removing the tube is much more complicated. The gag must be introduced, the strapping taken off, the string unhooked from the ear, and, while the one hand holds the string loosely, the index of the other hand feels for the string at the back of the child's larynx, hooks the index round this string, and pulls it out quickly with the tube, the other hand releasing the far end of the silk. This extubation can, in an emergency, be easily performed by one nurse, as the gag is self-retaining, and the nurse, after having fixed it, has both her hands at her disposal. Many doctors will let the nurse do this little operation under their supervision whenever there is a chance, so as to give her confidence and make her skilful to act quickly in critical times.

When, on wishing to withdraw the tube (passed through the mouth) the doctor finds that the string has been bitten through, the extubating instrument has to be used. The nurse's duties in this case are the same as for intubation, namely, immobilising the child, maintaining the head absolutely straight, and holding the gag. The doctor will insert the beak of the extubator into the lumen of the tube, make the jaws of the extubator grip the tube by pressing the lever on the handle with the right thumb, and withdraw the mounted extubator quickly.

In successful cases the tube is generally removed after two or three days, but the nurse must immediately boil it and get it ready for reintroduction, because when the tube is taken out children will sometimes behave in a most neurotic manner, and cough and choke until the tube has to be put back. Of course, in many cases the condition of the larynx requires the tube for a longer time, but in others the nurse can do a great deal to tide the child over the bad few minutes when the irritation caused by the withdrawal of the tube is very troublesome, and so enable the tube to be left out permanently. Play with the child and take its attention off its woes; with the doctor's permission, nurse it in front of the fire—as flat as possible; even a nice hot bath has sometimes been a great help and comforter. Several doctors have extubated under an anæsthetic successfully when repeated natural efforts of leaving out the tube had failed.

The doctor generally orders cases of intubation to be fed with the nasal tube. Patients with an intubated larynx swallow liquids—which are their chief nourishment—very badly, and the danger of some of the food finding its way into the lungs and setting up fatal complications is very great.

The nasal tube consists of an india-rubber catheter, a small glass connection, a short piece of india-rubber tubing, and a glass funnel. The catheter is lubricated with olive oil, and passed gently down the nostril. When it reaches the back of the pharynx the patient will, as a rule, try to retch, and this may result in the tube being forced into the mouth. The child will soon overcome this tendency, but we must always bear in mind the possibility of its occurrence, and inspect the mouth, to make sure the catheter is in its right place in the œsophagus, before pouring down the fluid. It is surprising how soon a child will get used to this form of feeding, and after the first disagreeable moment of feeling the tube in the nose will watch the proceedings with interest. The feeds are generally given four-hourly during the day and six-hourly during the night, the amount varying according to the age of child. It is well to point out here that the condition of the mouth of these artificially-fed children wants special care, and in addition to the routine cleaning frequent swabbing out with cold water will allay the child's thirst and make the poor hot mouth fresh and sweet. If the nasal tube is objected to, or additional nourishment is required, it is best to give it to the patient in the form of semi-solids: milk-jellies, arrowroot, Brand's Essence, etc., which he will swallow with less difficulty than liquid food.

One more important injunction. Never leave an intubated patient alone, even for one minute. The result might be disastrous.

*(To be concluded.)*

### The Nurses' Clinic.

### MINOR SURGERY IN DISTRICT NURSING. BY MISS M. LOANE.

A considerable part of a district nurse's duty consists of minor surgery, and if she is capable of performing it in an efficient manner she will largely increase her general influence among the poor (always an important matter when serious and infectious disease may have to be considered), soothe an amount of suffering which in the aggregate is enormous and in detail irritating and temper-wearing, and prevent small injuries from being aggravated into a state so hopeless that the most skilled surgeon will not be able to keep them from resulting in lifelong pain or deprivation.

It must be noted that whether the local regulations do or do not permit the district nurse to be in attendance where no doctor has been called in, her duties will be practically the same with reference to all work of this nature, as among the poor the doctor's supervision of unvarying chronic and minor surgical cases becomes merely nominal as soon as he has secured the regular attendance of a trained nurse.

The preparation for the skilled and, as far as possible, painless performance of minor surgery must begin with the mental and physical condition of the nurse herself. She must not only know precisely what she is to do, but she must keep hands and nerves under such control that she can carry out the work swiftly and surely. The preliminary arrangements for the work may be as slow as circumstances ordain, and as a rule the poorer the household the longer it takes to find and adapt the few indispensable articles. But the work itself, to be efficient, must be executed with the same unhurried rapidity, whether it has to be done in a first-class hospital, a luxurious private house, or a crowded tenement.

The nurse must be especially careful to keep her hands not merely free from cracks and roughness, but soft and supple, and her exact and precise control over them must never be injured by coarse housework or lifting heavy weights. Most people realise that if they had scrubbed a floor, blacked a grate, cleaned a few kettles, and polished several square yards of brasswork, and then turned to piano or violin, water-colour painting, or even fine needlework, they would find that they had temporarily lost the control of the hands necessary in all these pursuits. How, then, can it be otherwise if they turn from these violent efforts to minor surgery?

The necessary knowledge and the physical ability being taken for granted, the third essential is orderly method, and the district nurse, too suddenly released from hospital routine, or despairing of maintaining a strict system of procedure in cottage kitchens and attic lodgings, too often falls far short of what is easily practicable. A fourth essential is sympathy, and the recognition of how much suffering is caused by the mere anticipation of suffering.

The most common form of minor surgery needed in poor neighbourhoods is the right treatment of varicose ulcers. These ulcers are, unhappily, so common that I have heard doctors ask, "Not much work, I suppose? Just a few old legs?" and nurses in even more contemptuous tone protest, "I don't call that nursing!" Happily these ulcers, though common, are curable, even when the case is of long standing, has been previously neglected or mismanaged, and the sufferer is of advanced age. Not long ago I had a patient of seventy-eight, an extremely bad case. I attended twice daily. In four months she was decidedly better, and in six completely cured. Except that she was willing to remain in a recumbent position, all the conditions of her daily life were against her—bad food, bad air, and the semi-darkness of a courtyard room, while strictly local cleanliness was all that she would permit or I could achieve.

All the articles necessary for a surgical dressing must be collected and arranged. If the ulcer is severe and has been previously neglected the first thing to be done is to cleanse it by the application of warm boracic fomentations, applied twice daily as long as may be necessary. The innermost layer of the fomentation should be of lint, but the three outer ones can be of clean boiled rag. Oiled silk should be placed outside, then wool, and the whole kept in place by a bandage firmly but not tightly applied from the foot upwards. When the dressings are changed the wound must be well washed with a Higginson's syringe and warm boracic lotion. When

**THE NURSES' CLINIC**—*Continued.*

healthy granulations have filled up the wound boracic ointment spread on lint may be used, the lint being cut to the exact pattern of the wound. The essentials of cure are cleanliness, careful bandaging, and complete rest, with the leg raised so as to counteract the tendency to venous congestion. Of these the nurse will find the last the hardest to enforce.

Superficial burns are next in frequency, and for ordinary cases no dressing equals four layers of lint (the three outer ones may be clean rag) soaked in warm carron oil covered with wool, oilskin, and a bandage. If the surface of the burn is large the dressings must be laid on in strips, never in large pieces, as the removal of these would be painful or even dangerous. In district practice I have not found it advisable to wash the surface of burns under pretext of removing the discharge. Looking back on all the worst cases of burns that we have had I find that whenever this treatment was ordered cure has been slow and painful as compared with those instances when we were permitted to replace instantly the old dressing with a new one.

If hand or foot is burnt a plug of absorbent wool should be soaked in warm carron oil and placed between fingers and toes at the root. For the hand a cardboard splint well padded with wool will be found useful. Changes of dressing are generally bad, and should never be tried except by the doctor's express wish.

The modern plan of treating burns by simply applying one layer of lint or rag soaked in warm boracic lotion and making a wire cage covered with calico and affixed to the patient by tape strings, can easily be applied to hand, arm, foot, or leg, and is generally successful.

Abscesses until they break or are opened should be frequently bathed with warm boracic, and in the intervals covered with warm wool and a bandage. After an abscess has broken it should be well syringed with warm boracic lotion and then covered with a boracic fomentation, the dressing being renewed every four hours. As soon as the surface is ready to heal boracic ointment may be used. For carbuncles a carbolic compress 1-60 or 1-80 is very useful.

### The Waltham System of Nursing.

### A COMPLEMENT TO TRAINING SCHOOL METHODS.

This system of training nurses, which was founded by Dr. Worcester at Waltham, Mass., twenty years ago, has been called "Burdettism" by some whose strong point is certainly not precision of statement. A visit to Waltham, and a careful study of the whole system, reveals that in practice it amounts to a rebellion against those who hold that patients, whether in private houses or public institutions, who are suffering from disease or accident, should be regarded as cases and not as individuals. Dr. Worcester rightly holds that nursing is mothering, and he is striving to secure that no woman shall become a nurse through the Waltham School who is not, in fact, tender, kindly, and considerate, and who does not recognise in the patient a fellow human being who needs, and is entitled to receive, tender sympathy and consideration.

In order to attain his aim he has laid it down that hospital nursing is only one department of the nurse's training, and that the woman who has received nothing but hospital training, may, in fact, prove a most undesirable member of a household when one of its inmates is in need of a nurse's services. Dr. Worcester has, therefore, organised a plan whereby every probationer in the Waltham School is thoroughly taught (1) district visiting nursing; (2) the nursing of private patients in their homes; (3) the mothering of infants from their birth; (4) the care of chronic invalids and dying patients; (5) obstetric and midwifery nursing; (6) housekeeping, including its chemistry and dietetics, the purchase and testing of foods of all kinds, and the cooking and serving of invalid diets; (7) the importance of cleanliness in detail, and the particular application of asepsis to everything connected with the sick.

#### An Initial Difficulty Overcome.

At the Waltham School the course of training occupies four years. The first year is devoted to the preparatory course, an unique feature of the Waltham training established in 1894, when it proved a great innovation. It originated in the conviction that it is impossible by entrance examinations, or after a month or two's probation, to discover which candidates for nurse training are in reality well fitted to undertake it. All who are connected with the training of nurses know by experience that certificates of health and of excellent character sometimes prove valueless—first impressions do often prove deceptive—and consequently that a closer acquaintance and more intimate knowledge of every applicant is plainly needed before a prudent administrator can decide which of them all is most fitted to train as a nurse. All these difficulties disappear through the preparatory course at the Waltham School, which provides a thorough test of the probationer's capacity to be a nurse. Its object is by actual trial to discover the probationer's physical endurance, handiness, tact, and devotion to the service of the helpless. The tests are applied in the home work of the school, and in the district visiting nursing of the physically helpless. This combination of study and drill—of theory and practice—is the essential characteristic of the Waltham method. Although there has been a general movement among training schools in the direction of preliminary education, the majority of them still fail to apply the Waltham test, and so the product of several of them as expressed in the finished article leaves something to be desired.

#### The Preparatory Course.

The preparatory course has six branches: (1) domestic science; (2) housekeeping; (3) chemistry, anatomy, physiology, hygiene, materia medica, bacteriology, and medical chemistry; (4) district visiting, nursing of infants, convalescents, and chronic patients; (5) personal improvement; (6) the care of the outside of the body, or surface nursing. In practice we find that forty lessons are given to each student in domestic science, and that each has eight weeks of practice work in the kitchen, where they have to cook and serve each day for from thirty to fifty persons. The dietetic course aims to give the student an understanding of the economic, nutritive, and physiological values of food, and teaches how to plan dietaries for the healthy and the sick. The results are excellent as we can testify from personal experiences. In the general cooking the five food principles; *i.e.* water, mineral matter, proteids, carbohydrates, and fats, are studied with reference to their composition, sources, and methods of preparation. Each probationer has experience in marketing during her term in the kitchen, and the class is taken in small groups to the large markets where the different food products are shown—the cuts of meat pointed out, and their special uses described. This branch of instruction is directed by Miss Irene Ballantyne, a graduate of the Mary Hemenway department of household arts of the Framingham, Mass., Normal School.

The Waltham system further holds that nursing, after all, is largely housekeeping for the sick, and in accordance with this belief all the housekeeping work of the home is done by the probationer under the close supervision and constant teaching of the principal and her assistants. The class is divided into squads, each having its special part of the housework, and so a uniform training is given to every probationer. In Branch 3, Anatomy, etc., is thoroughly and exhaustively taught, whilst in branch 4 district nursing visits, under an instructor who is a graduate nurse, enable practical tests to be applied to the natural fitness of each probationer, and afford all the students most important lessons in the art of nursing. In this visiting nursing the class of probationers is again divided into squads, in order that each in turn may have practice in every branch of the work. Most of these nursing visits are to lying-in mothers and their babies. At each home the mother is bathed, her hair is brushed and braided; the bed is changed; her room is put in order; her gruels and broths made, and her baby washed and dressed. Observations of her condition are recorded on slips which the probationer takes to the doctor for his inspection and orders. When the probationer first enters the visiting nursing service the work is done by the instructor herself; then the probationer proceeds to do first one and then another part of the work under the instructor's supervision; then she visits alone, and while so engaged is visited and criticised by the instructor; or the latter may visit the patients after the probationer has left, to see that everything is well done. The patients are usually those of physicians who serve as instructors in the Waltham School.

In the 5th Branch, Personal Improvement, three lectures are given in the history of nursing and its higher meaning; four lectures on personal hygiene, and twelve lessons in reading aloud. Once a week for several months there is a class in voice culture, where special pains are taken to make each probationer use her voice rightly in speaking and in reading. Gymnastic exercises under the direction of the instructor in physical culture are a feature of this school. Throughout the whole life of the school and its work the constant effort is to bring out in each probationer her own best self, round off her angles, and so enable her to easily fit in with others in the work she has undertaken. Importance is attached to absolute neatness and punctuality, thoroughness in every duty, a cheerful obedience, and a ready recognition of the advantages these opportunities afford her for the formation of habits upon which successful nursing depends. In the 6th branch, Surface Nursing, for two hours three times a week for three months probationers receive thorough instruction in massage, in manicuring, and in the care of the scalp and hair. The surface nursing is in the charge of Miss Alice Kersting, the instructor. Altogether the preparatory course lasts for ten months; *i.e.* from October to July, when the student nurses have two months' vacation, and the same vacation is granted in each of the three succeeding years of training.

### The Three Years Subsequent Training.

In the year following the preparatory course the probationers begin their regular hospital training. Throughout the year they are at work in the wards of the Waltham Hospital and the Baby Hospital, where they are under the teaching supervision of the instructors of hospital nursing, of infant nursing, and of operating-room technique. In the third year the probationer passes on to the maternity and contagious diseases departments, and may spend a part of her time in the general wards also. A portion of this year may be devoted to service in the private practice of the physician instructors, and in visiting nursing. The instructors in visiting nursing are Miss Grace A. Reynolds and Miss Alice M. O'Neil. In the fourth year the probationers continue working in the various departments of the hospital and in district visiting and home nursing under the supervision of their instructors. It is claimed that the school gives an unusually large amount of didactic instruction in the preparatory and junior years in the attempt to teach the students beforehand what afterwards they must carry out in practice, as is customary in medical schools. At the end of the first two years the regular courses of class instruction end, and the subsequent training in the different departments is given mainly in actual nursing work. The educational principle underlying the Waltham system is its aim to arouse the intelligence of the nurses by making them first acquire a general knowledge of disease to the extent nurses ought to possess it, before they are given the actual nursing of patients suffering from any form of malady.

Midwifery training includes the preparation of the patient's bed for midwifery cases; how to obtain asepsis; to manage instruments and surgical material; to serve as surgeons' asistants; and how to work well together with the other nurses engaged on a particular case.

Senior probationers receive instruction by lectures in special branches of nursing at evening courses, which are taken down in shorthand, and copies are sent to such nurses connected with the school as may not be able to be present. The courses include the nursing of—(1) infants and children; (2) the nursing of women; (3) the nursing of the insane and of neurasthenics; (4) the nursing in cases of eye, ear, throat, and skin diseases.

### Its Popularity and Finances.

When the school was established in 1885 only local needs were considered, but the popularity of the Waltham nurses is extending the area of Waltham nurses' work indefinitely. The demand for the services of the student nurses in households so far exceeds the supply that it is not unusual to refuse a hundred calls a month. In the first year the percentage of employments to nurses ready for service was 60 per cent.; during the twentieth year the percentage of employments was 95 per cent. The school buildings cost $50,000, that is, £10,000, and consist of a furnished building that is well adapted to the needs of the school. It is used as a dormitory for the probationers, and as a refectory for the whole school. In addition it provides ample classroom and laboratory accommodation. There is a second building known as the Putnam Home, where graduate nurses reside, together with many of the junior and senior student nurses.

Financially the school has been a wonderful success. The tuition fees were first fixed at $100 for the first year, and $50 for each of the three subsequent years, making a total of $250. The present fees are $150 for the first year, $100 for the second year, and $100, in addition, being the cost of uniforms and outfit—making together $350, or £70, as the cost to each student nurse for her four years' course. A limited number are admitted to working scholarships, whereby students are enabled to pay their tuition fees by giving six months of extra service beyond the forty months required in the four years' course. By this latter arrangement the probationer enters three months earlier, and has her annual vacations reduced from two to one month. The school has been practically self-supporting, as the total sum received in voluntary contributions has not exceeded $7,000 during the twenty years; but there was a deficiency of about $1,000 last year, when the earnings of the school amounted to upwards of $15,000.

We have given a very full account of this school and its work to enable everyone to understand exactly what it aims at and accomplishes. An examination of its merits and shortcomings will constitute the Outlook article next week.

### Incipient Hip Disease.

### EXAMINATION QUESTIONS FOR NURSES.

The question was as follows: What symptoms in a child would cause you to suspect incipient hip disease necessitating medical advice at once?

### First Prize.

The following symptoms might be suspected as being due to incipient hip disease: Restlessness at night, especially if lying on one particular side. Probably at this time or during the night the so-called "night-cry" will be heard, and once heard it is never forgotten. A startling, short, sharp, piercing cry, and the patient is quite unconscious of it. He may be perspiring with some flushing of the cheeks, but there is no rise of temperature as a rule (at this stage). During the forenoon he will most likely seem very comfortable, but in the afternoon, and especially in the evening, he will show signs of irritability and restlessness, will not sit long in one position, and may complain of pain, usually described as "growing pains," in the thigh and especially the knee. It may be noticed on going to bed that the child limps and slightly drags the leg, with a tendency for the toes to point outwards or inwards. If looked at from behind one side of the pelvis may be somewhat higher than the other, only the toes on that side touching the ground. There may be some swelling and pain round about the joint, with enlargement of the glands in that region. If the knee be carefully flexed on to the abdomen, and the muscles fix (a kind of spasm), this is undoubtedly to prevent movement of the joint; there may also be some flabbiness and wasting of the gluteus muscles on the affected side. Lanoitan.

### Second Prize.

If the child had hitherto not made any complaints about himself, and had been apparently in a condition, but now commences to complain of pain in the region of the hip joint, or at the knee, where the acquired pain from the hip is felt; or, if he had previously been active in the use of his lower limbs, but now shows signs of a limp while walking, or has difficulty in the free movement of the hip joint attended with pain, I should carefully observe if there were any external signs either at the knee or hip to account for the pain. If one leg was the slightest amount shorter than the other, or if in the movement of the thigh by flexion the pain was increased, while by extending the whole limb by pulling it straight down with my hands just above the ankle the pain was decreased, I should immediately fear incipient hip disease, and should at once call in medical advice.

Ebor.

### Merits and Defects of the Papers.

"Lanoitan" wins the first prize with the best all-round paper. He mentions two very early and important signs, the pain in the knee and the piercing night cry. "Ebor" is successful in gaining the second with a good short paper, which shows some considerable observation. Several very good answers have been sent in, and four have been awarded Honourable Mention, but a large number of papers this month have been torn up because they bear evidence of being taken from handboks on nursing.

### Honourable Mention.

This is gained by "Bunny," "Bye," "Sunshine," and "Challie."

"Bunny" contributes a good paper, but goes beyond the question by entering on the subject of later symptoms. "Bye's" answer is very good, and came near to gaining a prize, but he must be a little more careful as to wording his paper another time, this one being somewhat obscure.

### Question for November.

How would you proceed to remove the coat and under garments from a patient who had a simple fracture of the right arm, and when the arm was sufficiently well to admit of a sleeve being used how would you re-dress him?

N.B.—This is a very simple question, but be careful as to the answer. The Examiner.

### Rules.

The competition is open to all. Answers must not exceed 500 words, and must be written on one side of the paper only, without divisions, head lines, or marginal notes. The pseudonym, as well as the proper name and address, must be written on the *same* paper, and *not on a separate sheet.* Papers may be sent in for 15 days only from the day of the publication of the question. All illustrations strictly prohibited. Failure to comply with these rules will disqualify the candidate for competition. Prizes will be awarded for the best two answers. Papers to be sent to "The Editor," with "Examination" written on the left-hand corner of the envelope.

In addition to two prizes honourable mention cards will be awarded to those who have sent in exceptionally good papers.

N.B.—The decision of the Examiner is final, and no correspondence on the subject can be entertained.

Any competitor having gained three prizes within the current year shall be disqualified from taking another until 12 months shall have expired since the first prize was gained.

### Royal National Sanatorium, Bournemouth.

The second annual "Linen Show and General View Day," inaugurated by the Ladies' Household Linen Association of the Royal National Sanatorium, Bournemouth, took place on Saturday last. The wards and corridors were prettily decorated with plants and flowers, and the whole building was thrown open from 3 to 5.30 p.m. The linen collected and purchased by the Association was on view in the women's corridor, and comprised no fewer than 557 articles. The Committee of the Association, taking into consideration the fact "that the patients of the Sanatorium, sleeping under open-air conditions, require especially warm clothing on their beds, decided that after purchasing such linen as was estimated would replace articles condemned during the current year, to spend the rest of money subscribed upon blankets and rugs for the patients' beds and outdoor shelters."

The balance sheet showed that the total value of the linen received from members was £22 9s. 10d., whilst subscriptions and donations amounted to £65 16s. A balance of £13 12s. 2d. in the treasurer's hands at the beginning of the year enabled the Association to spend altogether £100 3s. 10d. on the much-needed linen and blankets, and articles worth this sum were therefore on exhibition.

### To Nurses.

We invite contributions from any of our readers, and shall be glad to pay for "Notes on News from the Nursing World," or for articles describing nursing experiences at home or abroad dealing with any nursing question from an original point of view, according to length. The minimum payment is 5s. Contributions on topical subjects are specially welcome. Notices of appointments, letters, entertainments, presentations, and deaths are not paid for, but we are always glad to receive them. All rejected manuscripts are returned in due course, and all payments for manuscripts used are made as early as possible after the beginning of each quarter.

### Central Midwives Board.

### THE EXAMINATIONS.

A meeting of the Central Midwives Board was held on Thursday last week. There were present: Dr. Champneys (in the chair), Dr. Dakin, Mrs. Latter, Miss R. Paget, Sir William Sinclair, Miss Wilson, and Mr. Parker Young.

The question of printing the Rules of the Board in Welsh again came up for consideration, in consequence of a letter from Dr. W. Williams, County Medical Officer for Glamorgan, forwarding copy of a resolution of the Midwives Executive Committee of the Glamorgan County Council recommending that this course be adopted. The Board decided that the matter must be postponed until they had been able to get a consensus of opinions on the subject from the various Welsh County Councils who had been circularised by the Board many months ago.

A letter from Dr. James Wallace raised an important point. He asked to be informed what signification was to be attached to the words "under my supervision" in Form III. of the Schedule to the Rules regarding the signing of certificate of attendance on cases. The certificate runs: "Has, under my supervision, attended and watched the progress of not fewer than twenty labours, making abdominal and vaginal examinations during the course of labour, and personally delivering the patient."

It was proposed by Dr. Dakin that Dr. Wallace be informed that the meaning of the rule was that no one was competent to sign the certificate who was not present at the time when the candidate was complying with the terms of the rule. Mr. Parker Young opposed this strongly, urging that they were asking impossibilities, and instancing the way in which the certificates of medical students at the London hospitals were signed by the supervising doctor, who had not been actually present during the examinations of the patient. The words "under my supervision" should be interpreted liberally, and implied merely that the doctor had satisfied himself that the work had been done. Sir William Sinclair said that because the system with regard to medical students was a lax one, it was no reason why they should perpetuate it; it was a known fact that the average of deaths in cases attended by medical students was very high. Twenty cases was but a small minimum, and they ought to ensure that they were properly treated. Dr. Champneys said they must regard the matter from the point of view of the midwife, and must not place too great difficulties in the way of her obtaining her certificate. Finally, an amendment was carried, by four votes to two, that Dr. Wallace be referred to the rule and be informed that the responsibility rests with the person signing the certificate.

The Secretary reported that four additional examiners had been appointed. The number of candidates had increased in London to 353, as against 223 at the last examination. The examiners appointed were Dr. Hammond Bell, Dr. Gow, Dr. Rivers Pollock, and Dr. Drummond Robinson.

The consideration of the number and approximate dates of examinations to be held annually in London and the provinces then came up. At the Conference in London the feeling had been in favour of a bi-monthly examination, beginning in February each year. The Board dealt with the question of London first, and Dr. Dakin, seconded by Mr. Parker Young, moved that the examination be held every two months. Sir William Sinclair moved that the examination be held three times a year. His motion was not seconded, and Dr. Dakin's resolution was carried. The Secretary informed the Board that he had received replies on the matter from all the provincial authorised training schools, and the general opinion was that the examination should be held every three or four months. For the ensuing examination the number of candidates in the provinces was 118 only, as against 353 in London. A letter from Manchester was read, urging an examination every four months, but protesting that London and the provinces should be treated alike. Mr. Parker Young, seconded by Miss Wilson, moved that for the present the examination in the provinces be held every four months. Sir William Sinclair, seconded by Mrs. Latter, moved as an amendment that the provinces and London be treated alike. The amendment was lost. The fixing of the exact dates of the examinations, which are to be held early in the months specified, was left in the hands of the Chairman and Secretary.

The report of the Standing Committee of October 12 was adopted.

Miss Wilson moved: "That the accompanying form of directions to the Board's inspector be approved and issued to her for her guidance in the performance of her duties." Miss Paget seconded the motion. Sir William Sinclair thought that the Board assumed the competence of the inspector by the act of appointment, and that the directions were altogether superfluous, if she knew her duties. Exception was taken to some of the detailed suggestions, and it was decided to postpone the matter in order that members could send proposed alterations to Miss Wilson for her consideration.

In order to despatch the arrears of business it was decided to hold a Standing Committee on November 9.

### Association for Promoting the Training and Supply of Midwives.

### AN INCOME OF £1,000 A YEAR WANTED.

A meeting of the Council of the Association for Promoting the Training and Supply of Midwives was held on Friday last. The chair was taken by Mr. Arthur L. Leon, Hon. Treasurer, and among those present were Lady Brassey, Lady Priestley, Mrs. Charles Schwann, Mrs. Wallace Bruce, Mrs. Samuel Bruce, Miss Rosalind Paget, Miss Lucy Robinson, Dr. Mary Rocke, Miss Grant, Miss Gill, the Secretary, Dr. Boxall, and Mr. Wallace Bruce.

The election of new members of Council having taken place, Mrs. Wallace Bruce, as chairman of the Executive Committee, read a report of the work done since the last meeting in February. Two more provincial centres had affiliated—*i.e.*, Newport (Mon.) and Wells (Cambs.). A paper suitable for reading at mothers' meetings had been drawn up and largely distributed. Much correspondence with local authorities had been conducted, and erroneous statements in the Press had been corrected. In June the Central Midwives Board had altered the time for examinations from every four months to every three. This change the Association had protested strongly against, and had suggested as an alternative that the examinations should be held every two months. An important new departure in their work had been the lectures to existing midwives in London. The Education Committee of the London County Council had arranged for lectures, lent schools, and supplied all necessary plant, but had asked the Association to organise the audiences. There had been seven lecture centres last spring, at each of which thirteen lectures had been delivered at a fee of 1s., and with an average attendance of 15. Dr. Collie, the medical superintendent of these classes, in his report remarked upon the large number of births attended by these "bonâ-fide" midwives, one woman being responsible for nearly 600 cases a year, and yet she only missed two out of the 13 lectures. Evidence of the interest taken in the work was shown, Dr. Collie went on to say, by the fact that one lecturer alone procured for the members of her class, at their request, £20 worth of the appliances that the Act decided to be essential for their work. The information given in these classes on such subjects as suitable diet for infants, value of fresh air, and the importance of cleanliness, was retailed to the poor at a time when they most needed it. If it was assumed that each of the midwives attending these classes attended two births a week, it followed that 1,500 mothers would be visited by these women in the course of the next twelve months. The Education Committee, Mrs. Wallace Bruce said, were arranging for lectures at eleven new centres, beginning this week. This new departure would, she felt sure, prove of the greatest practical value. The grant of £100 to the East Ham Home had been continued; during the last nine months 505 cases had been attended and 10,121 visits to mothers paid. The Association were to be congratulated on possessing so excellent a midwife in charge as Miss Rabson. Twenty-four midwives had been or were being trained at a cost of £408, £80 of which was provided by the women themselves. Thirteen had been trained with a view to returning to work in their own districts. They had six more candidates ready to be trained, who were waiting for the necessary funds.

Mr. A. L. Leon then read the financial statement, showing that in 1904 they had received £235 in subscriptions and £630 in donations. All this money had been spent or pledged, and he urged the members of the Council to do their best to interest other people in the work, which would appeal to the hearts of thousands if they only knew about it. They had expended £60 in advertising the classes for "bonâ-fide" midwives, and this sum, he was of opinion, should be refunded to them by the Education Committee. They could not carry on their work successfully unless they had an income of £1,000 a year.

A resolution was proposed by Lady Brassey approving of the work done by the Executive Committee, and suggesting that in order to obtain more funds a large meeting should be held in the spring. The offer of her house for the purpose was gratefully accepted. Miss Paget remarked that the Central Midwives Board had decided to hold the examinations every two months.

An interesting report on the lectures was read by Dr. Mary Rocke, who particularly remarked upon the change in personal cleanliness, etc., that was to be observed in these uncertificated midwives, and referred to the great assistance at some of these lectures afforded by Miss Gill.

A suggestion was made that a systematic collection of funds should be organised, and that London should be divided into districts for this purpose.

A vote of thanks to the Chairman terminated the proceedings.

### Examination at Clapham Maternity Hospital.

The second public examination of the Clapham School of Midwifery took place on Saturday, at Clapham Maternity Hospital.

A three-hours' paper was followed in the afternoon by a *vivâ voce* examination and a clinical examination in the wards. Twenty-two candidates presented themselves, including those from distant provincial schools, the only condition required being that the candidate should have had not less than three months' training in midwifery, applications being accepted till within twenty-four hours of the examination. Of the candidates, seventeen passed, two with marks sufficient to earn "Distinction," while five failed to satisfy the examiners. The latter are medical women, experts in midwifery but unconnected officially with any special school of teaching. The next examination will take place on February 10, 1906.

### Incidents in a Nurse's Life.

Contributions to this column are invited.

### A KAFFIR AND A GENTLEMAN.

"Poor child! I'm afraid it won't be long," sighed sister as she put on her cloak and picked her way slowly through the mud to her tent.

It was 9 P.M., and I turned back again to my little patient, who lay so limp and helpless on the bed. She was a Kaffir girl, about twelve years old, who had been run over by a train, and both legs were horribly mutilated. A white child would have died outright, but she still lived, and whether she would rally sufficiently for the major operation which must inevitably be performed, and whether she could possibly survive the further shock of double amputation, were questions one feared to ask.

On the ground, near the bed, sat two bowed silent figures, her mother and brother. For hours they had crouched there, refusing all offers of food and drink with "My heart is sore; to-morrow I will eat bread," and except for an occasional shiver or sob from the mother they might have been carved out of wood. At ten o'clock the doctor came in, looked at the patient, shrugged his shoulders, ordered an ether hypodermic injection every two hours, and went away saying, "I give you *carte blanche*, sister; she will be gone before morning." I feared that the child would resist the treatment; but one word to her brother and he was beside the bed explaining in low gutturals to the sufferer that "no harm, but good, would come," after which he gently held her arm while I obeyed the doctor's order.

As the night wore on the child became delirious and restless, and, to my dismay, worked all her bandages loose, displaying the terrible wounds. Here, again, the Kaffir boy came to my assistance. Throwing off the tattered blanket he had wrapped round his shoulders for warmth, he washed his hands and arms as he had seen me do, and rendered help in the most dexterous manner, handling the poor limbs so tenderly and skilfully. It was, nevertheless, a long dressing, and the patient so collapsed that I gave a rectal stimulant to alternate with the ether injection, and so the feeble flicker of life was kept going.

When day began to break a low murmur of voices outside the hut told of the advent of other friends from the neighbouring kraals. The mother then rose, wrapped her blanket more closely round her, and stepped outside to join them; the brother still crouched by the bed gazing with dumb anguish at the sufferer, who now lay with scarcely a movement. When daylight filled the room I extinguished the lamp and opened the door wide. Suddenly a cry of "Ai, ai!" rang through the room. I turned. The little girl, with superhuman effort, had raised herself in bed and was groping feebly in the air with trembling hands; then as the brother and I both sprang forward, a dusky pallor spread over her face, and she fell back dead.

It was only an ordinary incident, no doubt, but the passionate, yet restrained, grief of the lad was not for an outsider to witness. I beckoned in the mother, closed the door, and left them.

### Presentations.

Essex and Colchester Hospital.—A handsome silver inkstand, pen, and paper cutter, with engraved monograms were presented to Miss Davies, the matron of Essex and Colchester Hospital, by the past and present nursing staff on the occasion of her departure to the Royal Hospital for Consumption, Ventnor. She was also the recipient of an illuminated address with a painted picture of the hospital, together with a purse of gold, from the committee, doctors, and many friends in the town of Colchester. Several other gifts were given her by her personal friends. During Miss Davies tenure of eight and a half years many improvements have taken place in the hospital, as well as the building of the now nice nurses' home.

### A Book and its Story.

A SOUL'S AWAKENING.

Mrs. F. Reynolds has founded her new novel on incidents in the lives of members of her own family, belonging to a former generation. "A Quaker Wooing" presents with accuracy, and in vivid contrast to the easy-going standard of manners that prevailed at the period, an attractive picture of a Quaker household in the eighteenth century, where ordered calm and a simple rule controlled the life of its members. The story is one that has to do with the families of two North-country squires whose properties adjoined. The following passage explains their relations: "Fowlds House and West Close lay in neighbouring parishes and about eight miles apart—a considerable distance in those days, when roads were far from good and none too safe on dark winter nights at all events. Yet the two families had been of old on the best of terms, and more than one Ackroyd-Holte marriage had served to cement the bond. Ackroyds and Holtes had fought side by side in the Civil War and alike pawned jewels and mortgaged lands for an ungrateful Sovereign. Later on came the Restoration." The Holte of the time being a student and scholar did not follow the family traditions. The gay life of the Court of the Second Charles had no attractions for him. He married a Puritan maiden, and from this date apparently a change came over the succeeding generations of Ackroyds, for when the story begins they had drifted from Puritanism into Quakerism, and the hero, John Ackroyd, is a staunch member of the Society of Friends. The Ackroyd family had but two surviving members—John Ackroyd and his blind mother—and they figure prominently in the story of "A Quaker Wooing."

The prettiest scenes are those connected with the gentle mother and her devoted son. Their neighbours, the Holtes, had an only daughter, Audrey, who was an acknowledged beauty. John Ackroyd, although he had been to Oxford and had travelled under the escort of a tutor, was a very different being to the men who frequented West Close, Audrey's home. He was known as "The Quaker Squire," a name which conjured up a curious anomaly of associations. As children he and Audrey had been playfellows, but since then they had hardly met until the day when the story begins, which was the occasion of a village feast, when the Ackroyd mansion of West Fowlds was thrown open, with its grounds, to the neighbourhood. Among the guests was a party from West Close. Audrey, bound on conquest, was there. It was the day when ringlets were part of the equipment which every woman carried as an aid to her charms. This is Audrey as she appeared at the revels. "Her hair, worn in a profusion of ringlets, was in startling contrast to her unusually dark eyes. It was dark brown, with glints of gold in the light and tinges of bronze in the shadow . . . crisped by the sunshine or ringed with damp when the wet wind blew over the marshes from the distant sea; it was always adorable—a fit setting for the perfect face of Audrey Holte. Living in an atmosphere of adulation she was not spoilt by it, for her inclinations and tastes were towards the happy country life and its simple joys, and not for the gaieties provided by town life and the circle of the Court. Better, worlds better, Audrey loved her beautiful country home, with its shady gardens and smooth-cut lawns, its yew hedges trimmed in fantastic shapes, its Dutch pleasance, all in orderly lines and trim rows, and the sunny kitchen-garden, where the clustering roses disputed possession of the mellowed brick walls with blushing peaches.

Untouched as Audrey's heart was at present she yet cherished certain definite ideas as to her future deportment, when she of her own free will should make choice of a lover herself. "Some day she would honour all by choosing *one*. Meanwhile were not men's hearts made to be played with? And just now the careless beauty, sighing for fresh hearts to trample, had demurely set her dainty foot upon a newly-surrendered trophy." The latest trophy is, of course, the Quaker Squire. "Since his father's death, some years earlier, the young squire, as he was still called, had lived on unmarried, caring for his widowed mother and administering the affairs of his large estate, as all agreed, with a wisdom and prudence beyond his years. Perhaps it was this early responsibility so manfully shouldered, perhaps also in part his Quaker training, that had given John Ackroyd a dignity and gravity of bearing that differed from the elaborated manners imported from France of the other young men of his age and rank."

After their meeting at the revels Audrey and John Ackroyd were constantly together. They met out riding, when Audrey, accompanied by a discreet groom, who had served her parents for many years, took her morning rides. One day as she was passing the entrance to Fowlds House she met its owner, who begged her to come in and see his mother. "Audrey . . . followed in silence through the massive hall with its wide fireplace, where even on this sunny May day a couple of logs smouldered on the wrought-iron dogs, into an inner chamber, where her host bade her be seated while he ordered some refreshment and ascertained if his mother were ready to receive a visitor." Left alone Audrey looked round on things that had not met her eyes since childhood with some curiosity. In the apartment in which she found herself the first thing that impressed her was a certain restful peace and extreme quietude. After a few moments of waiting there seemed something oppressive in the solemn atmosphere of the room. . . . "What a death in life!" she murmured as she got up and crossed the room." Upon the return of John Ackroyd she followed him to his mother's room. He reminds her that she is blind, a fact which Audrey had forgotten for the moment. "As Audrey stepped from the darkness of the passage into the room she was almost dazzled with a flood of golden light . . . and in the midst of the radiance sat the most beautiful old lady the girl had ever seen. She was leaning forward in her deep armchair, her fingers busy with some knitting. Her dress was of dove-coloured silk, very soft, fine and shimmering in the sunlight . . . a close-fitting cap of snowy muslin concealed the greater part of her silvered hair." To Audrey this picture of perfect old age was a revelation. The vision of another world where calmness and holiness reigned. It was in soothing contrast to some specimens of old age among her own circle. "The head was turned and the hand outheld in welcome, though the Quaker lady could not see her visitor. In spite of this it was at that very moment Audrey realised she was in the presence of the blind. The grey eyes turned towards the light were dim and soulless, their light was too evidently darkened. A minute later John had placed Audrey a chair close to his mother's side and had retired, leaving the young life and the old alike rapt about in sunshine." From these extracts of a book perfectly free from any objectionable element our readers can judge, we hope, of its claim on their further attention. Where there is a difficulty in finding books that are good without being dull, of the kind that elder girls can read, "A Quaker Wooing" will stand the test of a trial. It is an improvement on other books by the same author. The interest is concentrated on the hero and heroine, and in spite of rather too many "Quakerisms" the development of their romance moves both on natural and winning lines.

* "A Quaker Wooing." By Mrs. F. Reynolds. (Hutchinson and Co. 6s.)

### Appointments.

[No charge is made for announcements under this head, and we are always glad to receive and publish appointments. The information, to insure accuracy, should be sent from the nurses themselves, and we cannot undertake to correct official announcements which may happen to be inaccurate. It is essential that in all cases the school of training should be given.]

Brentford Union Infirmary, Isleworth.—Miss Edith Kitton has been appointed sister. She was trained at Whitechapel Infirmary, where she has since been staff nurse.

Basingstoke Isolation Hospital.—Miss Clara E. Wragg has been appointed matron. She was trained at the Monsall Hospital, Manchester, and the Royal Hants County Hospital, Winchester, where she has since been night superintendent.

Bristol Nurses' Institution and Nursing Home.—Miss J. M. McHardy has been appointed lady superintendent. She was trained at King's College Hospital, London, and has since been sister at the Princess Alice Hospital, Eastbourne, day sister and night superintendent at the Staffordshire General Infirmary, and lady superintendent of the Leicester Institution of Trained Nurses.

Gloucester General Infirmary.—Miss F. Christophersen has been appointed theatre sister. She was trained at the Preston Royal Infirmary, where she was subsequently theatre charge nurse. She has also been sister in charge of ward and theatre at the Birkenhead Borough Hospital.

Isolation Hospital, Selby.—Miss P. Pauline Crouch has been appointed charge nurse. She was trained at the Royal Infirmary, Manchester, where she subsequently held the posts of staff and theatre nurse. She has also been staff nurse at the Convalescent Hospital, Cheadle, and the City Fever Hospital, Leeds.

Novers Hill Hospital, Bristol.—Miss R. Crighton has been appointed night sister. She was trained at the Western Infirmary, Glasgow, where she has since been holiday sister.

Rotherhithe Infirmary.—Miss Mary Moreland has been appointed charge nurse. She was trained at Croydon Union Infirmary, and has since done private nursing.

Southern Hospital, Manchester.—Mrs. A. E. Firth has been appointed sister. She was trained at the Stanley Hospital, Liverpool, and Manchester Maternity Hospital. She has been sister at the Jessop Hospital for Women at Sheffield, and has done private nursing. She holds the certificate of the Central Midwives Board.

Winsford Hospital, Cheshire.—Miss Edith Fulford and Miss E. Fenton have been appointed charge nurses. Miss Fulford was trained at Salop Infirmary, and has since done private nursing. Miss Fenton was trained at the County Antrim Infirmary, Lisburn.

York Home for Nurses.—Miss Margaret Morgan has been appointed superintendent. She was trained at the London Hospital, where she was afterwards sister. She has since been matron of Addenbrooke's Hospital, Cambridge.

### Death in our Ranks.

We regret to hear of the death, from tubercular peritonitis, of Miss Martha Anne Clayton, aged thirty-three, at her home in Newtown. She was trained at the Marylebone Infirmary, and was afterwards district nurse at Bolton, and sister at St. Pancras Infirmary.

### Everybody's Opinion.

[Correspondence on all subjects is invited, but we cannot in any way be responsible for the opinions expressed by our correspondents. No communication can be entertained if the name and address of the correspondent are not given as a guarantee of good faith, but not necessarily for publication. All correspondents should write on one side of the paper only.]

### MINOR NURSING AMENITIES.

"The Elm" writes: I am sorry that nobody has written anything about the article entitled "Minor Nursing Amenities: a Matron as Patient." Being a private nurse of some years' standing, I have heard several complaints of the casual way in which the patients are treated in hospitals, in comparison with the dusting and sweeping of wards, etc. I hope that many matrons, sisters, and nurses have read the Matron's article and taken it to heart. It would perhaps be better if a few of them could be laid aside for a while, if only to experience the pleasure of their own, or somebody else's, severe and unnecessary regulations. I am sure they would go back to their wards "sadder but wiser women."

### DISTRICT NURSES AND MATERNITY WORK.

"Puzzled" writes: I am a nurse, trained in a large provincial hospital for three years, and have worked in other hospitals. I have been a private nurse, and also for some years a district nurse. I have always understood that nurses for maternity work ought not to go to any case that might be injurious to their patients. Now I find that nearly all district nurses take maternity cases. In the last three weeks there have been very few advertisements in The Hospital in which it has not been specified that the applicants must hold the certificate of the Central Midwives Board. Should nurses attend and dress all kinds of wounds, old sores, tuberculosis, and cancer cases, and then attend a maternity patient? In a district in which I worked for some years, one of the nurses was employed for maternity work only. She was often up all night, but could arrange to rest during the day. That would have been impossible if she had had the ordinary district work as well. I should like to know the opinion of other nurses, and if the medical profession approve of the combination.

[The combination to which you refer is distinctly to be condemned.—Ed. The Hospital.]

### MATERNITY NURSING IN SOUTH AFRICA.

"A Nurse in South Africa" writes: I hear through a reliable source, the matron of a public institution employing nurses in Cape Town, that since the passing of the Midwives Act many maternity nurses have applied to her for other work on the plea that they have come from England and can get no maternity work here. In connection with another institution which does employ maternity nurses I know that for two years and more several well-thought-of and capable maternity nurses have been seriously out of work, while maternity nurses working on their own account unsupported, have told me that whereas formerly plenty of work was to be had without seeking it, now it is most necessary to look patients up. Numbers of maternity nurses are now trained in the colony, and a very large number of home-trained general nurses now in this country have full maternity qualifications, so that there is no opening out here as far as it is possible to judge for maternity nurses any more than for general nurses. I was surprised to see in a nursing paper that Princess Christian is interesting herself in the training of midwives for Kimberley, and I fear that the situation out here in the nursing field can hardly yet be thoroughly understood in the nursing world at home.

### PRESENTS AT CHRISTMAS.

"M. R. N. P. F." writes: Will you allow me space in your valuable paper to make an appeal to the various matrons of hospitals to put a stop to the very great tax that Christmas presents have become. What originally must have been a pleasure has now become a mere compulsory gift, and in most cases the nurses, sisters, etc., are quite unable to afford so many presents as are expected from them. I have been a sister for eight years and have spent Christmas in three or four different hospitals, and everywhere I hear the same cry, "We can't afford it and yet feel obliged to give." The sisters are expected to spend money in decorating their wards, and in some have to help to provide an entertainment. Then presents to nurses, servants, porters, and possibly to each other, besides a handsome general gift to the matron, assistant matron, and others. When all this is done they cannot remember their home people and personal friends at all. In the case of the nurses it is almost worse, as their salaries are perfectly inadequate to cope with the drain on them at Christmas, and they have to appeal to their own relations, who possibly are not in a position to help. I feel sure that if matrons would only think of the matter they are the people most able to put a stop to the tax, and their nursing staff would be only too thankful to them for so doing.

"One Who Knows" writes: As the season of Christmas approaches, and many are looking forward to a "good time," may I put in a strong appeal for those to whom Christmas is a season of increased anxiety and an added burden not easily borne? I refer to the custom of present-giving in our hospitals among the matron, sisters, and nurses, which has reached such a pitch that the poor nurse finds herself at last bankrupt, without sixpence to buy even a trifle for some dear one at home, to whom giving was not a duty but a joy, and who henceforth must give place to the last newcomer, or to some acquaintance of a few days' standing. Here is the case put plainly: each sister must subscribe handsomely to the matron, who, in her turn, must give to all, each sister to her fellow sisters, and sisters and nurses alike to the whole staff, including wardmaids, porters, gatekeepers, cook, and hall-boy, etc. In addition, each sister is responsible for the decoration of her own ward, involving plants and flowers (always costly at such a time). She is expected to do her share in supplying the patients' Christmas-tree, often also sharing in a tea or entertainment for the patients. Unless a nurse wishes to be boycotted altogether, she is compelled to join in this wholesale taxation. Can nothing be done to stop this state of things? Can any of your readers suggest a way out? If so, he would earn the gratitude of everyone on the staff, from the matron down to the newly-joined "pro.," for all feel it; but because no one will stand out and denounce the system, nor propose a remedy, the intolerable evil remains all the more grievous that it is a hidden and secret one.

### THE LONG VEIL.

"M. R." writes: In last week's Hospital a "Regular Reader" suggests that only hospital nurses be permitted to wear the long veil. I should like to add a suggestion that the authorities should not allow their probationers to wear any kind of uniform until they have proved themselves suited for the work to which they are so anxious to show the outside world they belong. I have always considered the long veil as useless and expensive, but admitting that it seemed becoming to some, after nearly four years in hospital, when I started private nursing I arrayed myself in what I considered a desirable outdoor uniform, a neat well-cut circular cloak, and a pretty little bonnet with a long silky gossamer veil, all in a charming shade of dark blue. I must admit that the veil looked nice hanging in long graceful folds down my back—indoors. But out of doors, in a breeze, it was far different! I invariably pulled it round my neck and tucked it into my cloak, where at worst it made an ugly bulge on my chest, but sometimes it would suddenly escape from its temporary resting-place and affectionately hug a lamp-post or anything it came in contact with. In crossing a road it used to get across my eyes or frighten horses by being blown suddenly in their way, three times causing me to be nearly run over. Then, I fear, I looked hardly sane, in a high wind, with that veil standing perfectly upright about a yard and a half, and my arms making frantic endeavours to get it down! So I decided to dispense with it, but as the bonnet was not one I could wear without a veil I had to send to town for one. In the meantime, as if the thing were possessed, twice more that horrid veil got me into trouble. One day I was on the parade at Cowes. Suddenly a strong breeze came and carried my veil to the left, and when I tried to pull it back I found that it had settled itself on the face of a tall yachtsman with a big tawny moustache which looked very funny through the gosammer; and helplessly I watched him trying—oh, so gently—to disengage himself from the filmy covering. I was afraid to move in case my bonnet was pulled off my head. However, with the laughing help of one or two other yachtsmen "my prisoner," as he styled himself, was set free, and I quickly departed into the town for my shopping. Coming back I was walking very quickly downhill, when my poor little bonnet was suddenly pulled right off my head from behind. Or course, my white strings in front still held, but I felt awful. Whatever had happened this time? I soon found out. My veil had caught on a hook with some garments hanging outside a shop. I quickly untied my bonnet strings and went inside the shop, when my bonnet was released and brought to me with a big rent in the long veil. I put it on, twisted the veil so that the tear did not show, and pinned it very firmly inside the front of my cloak. When I got home that veil was just put on the kitchen fire. Needless to say I have never worn one since, and do not intend to.

### Novelties for Nurses.

(By Our Shopping Correspondent.)

### PERI LUSTA.

Peri Lusta is a beautiful thread which has exactly the appearance of silk, whilst being much stronger and less expensive. It is made in all colours and thicknesses, and in varying twists which render it suitable for all purposes of fancy needlework, where silk or cotton threads are used. The manufacturers of Peri Lusta have issued a very nice handbook, which contains most useful hints for work in which Peri Lusta can be employed. It can be procured by post for sevenpence from the Messrs. Blatchley and Ashwell, 19 Ludgate Hill, London, E.C.

### THE OLD TIME GARDEN.

The Old Time Garden is the name of a pretty little booklet which has been prepared by Scott and Bowne, the manufacturers of Scott's emulsion, at 11 Stonecutter Street, London, E.C. It is offered to nurses to distribute amongst their little patients, by whom it will certainly be received with pleasure. The Old Time Garden is a charming tale for children, prettily illustrated with coloured pictures. The advertisement does not detract at all from the book, though it should certainly induce its readers to try the emulsion.

### TRAVEL NOTES AND QUERIES.

By our Travel Correspondent.

Books to be Read About Rome.—Hare's "Walks in Rome" and "Days near Rome," Mrs. Minto Elliott's "Roman Gossip," Hawthorne's "Transformation," Ouida's "Ariadne" and "Friendship."

Outfit for Egypt (F. F. H.).—Take all underclothing of light wool. This is a safeguard against chills contracted in the cool evenings and in passing from sunlight to shade. Two tailor-made coats and skirts, also woollen, but of different thicknesses, one quiet looking but well made black dress for evening wear, and one thinner dress of Tussore silk or some such material, will, I think, be all you need for the entire trip, as you naturally wish to limit your luggage. You will most certainly need a shady hat with a veil to protect the neck. A rather smarter one for dressy occasions, and a quiet felt or straw for travelling. Shops are expensive in Cairo, so take with you a few necessary medical comforts, also a pair of smoked pince-nez as a protection from the glare.

Rules in Regard to Correspondence for this Section.—All questioners must use a pseudonym for publication, but the communication must also bear the writer's own name and address as well, which will be regarded as confidential. All such communications to be addressed "Travel Correspondent, 28 Southampton Street, Strand." No charge will be made for inserting and answering questions in the inquiry column, and all will be answered in rotation as space permits. If an answer by letter is required, a stamped and addressed envelope must be enclosed, together with 2s. 6d., which fee will be devoted to the objects of "The Hospital" Convalescent Fund. Ten days must be allowed before an answer can be published.

### Notes and Queries.

**REGULATIONS.**

**The Editor is always willing to answer in this column, without any fee, all reasonable questions, as soon as possible.**

**But the following rules must be carefully observed.**

**1. Every communication must be accompanied by the name and address of the writer.**

**2. The question must always bear upon nursing, directly or indirectly.**

**If an answer is required by letter a fee of half-a-crown must be enclosed with the note containing the inquiry.**

*St. Bartholomew's Hospital.*

(31) I should feel very grateful if you would tell me what subjects are necessary to pass the elementary examination for entrance into St. Bartholomew's Hospital as probationer. —*Midge.*

A would-be probationer, besides satisfying the examiners that she possesses general intelligence and has received a fair education, must have an elementary knowledge of (1) the names of the bones of the skeleton; (2) the structure and mechanism of the following points: shoulder, elbow, wrist, hip, knee, ankle; (3) the general situation of the viscera of the thorax, abdomen, and pelvis; (4) the course of the circulation of the blood; (5) the names of the various parts of the alimentary canal; (6) the principal parts of the nervous system; (7) the composition of the air; (8) the structure and general use of thermometers; (9) the signs and terms commonly used in prescriptions. They have also to pass a personal medical examination.

*Dispenser.*

(32) I have recently qualified as a dispenser. Can you suggest the best way of securing a post with a doctor?—*V. F.*

By advertising or answering advertisements in the medical and nursing journals.

*District Nurse.*

(33) Will you kindly tell me where I could get training for district nursing, and whether you think a general training is indispensable? And can you tell me what London hospital would grant a certificate for two years' general training?—*E. S.*

Your best plan would be to write to the General Superintendent of Queen Victoria's Jubilee Institute for Nurses, 120 Victoria Street, London, S.W.; but general training is indispensable. Few recognised training schools nowadays grant certificates for less than three years' training, but you could be taken as a paying probationer for two years at many hospitals, and would, of course, be entitled to a formal testimonial to the effect that you had received two years' training. See "How to become a Nurse," published by the Scientific Press, Limited.

*In-growing Toe-nail.*

(34) May I ask your advice about an in-growing toe-nail of very long standing? The nail was removed once (about ten months ago), and has grown again as bad, if not worse than before. . . . May I add that I read with great interest the "Notes and Queries" every week? It is a great advantage having the question put in the column with the answer, thereby giving others whom it may interest the benefit of the query.—*M. E.*

You can only consult a good surgeon.

*Probationer.*

(35) I have a great desire to become a nurse. I am very strong and healthy, domesticated, and well educated, but I am just turned 33. Is it possible to get into any hospital as a probationer? I notice most advertisements make 30 the age limit.—*Yorkshire.*

You would find it easier to gain admittance, especially at first, as a paying probationer. But if you are not able to do this the Royal Free Hospital, Gray's Inn Road; the Great Northern Central; the Poplar and Stepney Sick Asylum; the Holborn Union Infirmary, and others (for which see Burdett's "How to become a Nurse") take probationers till 35.

**Handbooks for Nurses.**

| | Post Free. |
|---|---|
| "A Handbook for Nurses." (Dr. J. K. Watson.) ... | 5s. 4d. |
| "Nurses' Pronouncing Dictionary of Medical Terms" | 2s. 0d. |
| "Art of Massage." (Creighton Hale.) ... ... ... | 6s. 0d. |
| "Surgical Bandaging and Dressings." (Johnson Smith) | 2s. 0d. |
| "Hints on Tropical Fevers." (Sister Pollard.) ... | 1s. 8d. |

Of all booksellers or of The Scientific Press, Limited, 28 & 29 Southampton Street, Strand, London, W.C.

### For Reading to the Sick.

THE SHADOW OF THE CROSS.

"We turn unblessed from faces fresh with beauty,
Unsoftened yet by fears,
To those whose lines are chased by pain and duty
And know the touch of tears.

"The heart whose chords the gentle hand of sadness
Has touched in minor strain,
Is filled with gracious joys, and knows a gladness
All others seek in vain.

"How poor a life where pathos tells no story,
Whose pathways reach no shrine,
Which, free from suffering, misses, too, the glory
Of sympathies divine!" *Anon.*

Suffering should always make us better and give us new skill and power; it should make our hearts soften, our spirit kindlier, our touch more gentle; it should teach us its holy lessons, and we should learn them, and then go on with sorrow's sacred ordination upon us to new love and better service. It is thus that lonely hearts find their sweetest, richest comfort. Sitting down to brood over our sorrows, the darkness deepens about us, and our little strength changes to weakness; but if we turn away from the gloom and take up the task of comforting and helping others the light will come again, and we shall grow strong.

In a sick-room there was a little rose-bush in a pot in a window. There was only one rose on the bush, and its face was turned full toward the light. This fact was noticed and spoken of, when one said that the rose would look no other way but toward the light. Experiments had been made with it; it had been turned away from the window, its face toward the shadow of the interior, but in a little time it would resume its old position. With wonderful persistence it refused to face the darkness, and insisted on ever looking toward the light.

The flower has its lesson for us. We should never allow ourselves to face toward life's glooms; we should never sit down in the shadows of any sorrow and let the night darken over us into the gloom of despair; we should turn our face away toward the light and quicken every energy for braver duty and truer, holier service.

The problem of all true living is not to miss pain or trial, but in all experiences, however hard or bitter, to keep our heart ever sweet and our ministry of good and helpfulness ever uninterrupted. The keenest suffering should make us only the gentler in spirit and send us out to be yet more loving and thoughtful—a benediction to every one we meet.

*Dr. T. Miller.*

It is because, let man do what he will,
Joy-light requires its sorrow shadow still;
It is because away God will not throw
His labour, letting His dear children go
Robbed of the needful discipline of woe.

*H. S. Sutton.*

